# Dr. Waters’ Case of Anomalous Periodical Ovarian Tumour

**Published:** 1842-10-01

**Authors:** Charles O. Waters

**Affiliations:** Broome Co., N. Y.


MEDICAL EXAMINER.

NEW SERIES.

No. 40.] PHILADELPHIA, OCTOBER 1, 1842. [Vol. I.

### *Case of Anomalous Periodical Ovarian Tumour.*

By Charles O. Waters, M. D.

(Communicated by Professor Dunglison.)

Mrs. M——, aged about 43, enjoyed uninterrupted good health till about the 18th year of her age, when great menstrual derangement, almost amounting to suppression, supervened. This derangement seems to have originated from sea-bathing, during the period of the catamenial discharge and at other times. The patient, from a feeling of delicacy too common in such cases, kept her disease as profound a secret as possible. About this time she was married, and it was not until some years after that, worn down almost by a profuse and obstinate leucorrhœa, which followed soon upon the menstrual derangement, and was finally attended with considerable prolapsus uteri, she laid her case fairly before her physician. Under his treatment the leucorrhœa was, I believe, entirely cured, and the prolapsus remedied by means of a supporter, which has been worn up to the present time. It does not appear, however, that the catamenia were *ever* rëestablished; at any rate they have not appeared for the last eight or ten years, and the patient, naturally of what is sometimes called the "nervous" temperament, has of late years laboured under excessive nervous impressibility. Some six or eight years since she began to suffer much from the frequent recurrence, in the præcordial region, of a pain very much resembling the pain of that anomalous disease, "angina pectoris." This being attended with dyspnœa, considerable ascites, &c., she was attended by several physicians in turn, and treated for pericarditis, "dropsy of the heart," &c., under all which no material amendment was perceptible. These spasms of pain becoming considerably aggravated, and being attended with considerable determination of blood to the head, at such times as the catamenial evacuation should naturally have occurred, monthly venesection was resorted to about two years since, and at the time of my acquaintance with the patient—about one year ago—had become an established habit. These monthly bleedings being undoubtedly injurious in their ultimate effects upon persons of such nervous impressibility, I am trying to discontinue them, and procure relief by the use of other means.

With these preliminaries, I come now to the facts from which I think the case derives its chief interest. The patient seems for some time previous to have suffered much from pain in the left iliac region, but it was only about two years ago—at which time she fell into the hands of my former partner, Dr. S. B. Hanford—that a tumour was discovered, *evidently rising from the left iliac region, and occupying at least one-half of the abdominal cavity.* It is not probable it had attained this size in a *very* short space of time. The breasts of the patient were enlarged, indurated, and very painful particularly the left,—and she had very much the appearance of a woman far advanced in pregnancy. I regret that I am unable to state the particular course of treatment to which the patient was then subjected, but mercurials and the various preparations of iodine were used both internally and externally. Diuretics and cathartics were also administered, and a strict course of diet and regimen entered upon.

Whether as a result of the treatment adopted or not I am unable to say, but about two months afterwards there occurred, *viâ vaginæ*, a sudden and profuse discharge of "a thick, yellowish, and ropy substance," filling two ordinary chamber vessels. The tumour, as examination showed, disappeared during this discharge, and after its cessation the left ovary could be felt nearly in its usual place, and about the size of a common butternut. This was in the fall of 1840.

During the winter the patient was tolerably comfortable under the use of the hydriodate of potash, laxatives, and diuretics.

The ensuing summer was spent mainly in the city of Troy; and in the month of September, 1841, she came under my medical care—ill health having compelled her former attendant to retire from practice. The ovarian tumour had now nearly regained its usual size; her breasts were swollen and painful; her stomach was very irritable; appetite poor; considerable anasarca, and much functional derangement of the heart, though no evidence, upon auscultation, of organic disease of that organ. The tumour was very painful, which, aggravating the previous nervous irritability, gave the patient but little rest night or day. I commenced the use of the following prescription:—

℞. Pulv. Camphoræ, ʒss.

Pulv. Opii, ℈j.

Ung. Hydrargyri Fort., ʒij.

Iodini, ʒj.

Axungiæ, ℥ij.

Ung. fiat.

Use two or three times a-day upon the tumour. Also,

℞. Gambogiæ, gr. vj.
Mit. Chlorid. Hyd., gr. j.
Jalapæ, gr. iv.

To be taken every other day.

Under this treatment the tumour enlarged but little, if any, but still continued painful. The purgative mixture was continued about ten days, when anodynes were resorted to more freely—the bowels being kept open by the use of Sulph. Magn. and Bi-Tart. Pot. The external application was continued.

About the tenth day the purgative mixture was repeated, and on the ensuing night a second discharge occurred, *by way of both vagina and rectum*—similar in every respect to the matter first discharged—but somewhat less in quantity. The change in the personal appearance of the patient was surprising. I now commenced the use of eutrophics—other than the preparations of iodine—for the purpose of restoring tone to the system generally; and of diuretics, to reduce the ascites and anasarca.

Under the use of these the patient's health, up to the month of April last, was better than it had been for many years before. The præcordial pain still, however, continued, and was relieved as before mentioned, and I had strong hopes that our old acquaintance, the tumour, had disappeared for ever. At that time, however, the pain in the left iliac region returned, and, after a short time, I perceived the left ovary enlarging for the *third* time. This it continued to do, attended with the same severe symptoms as marked its former growth, until about the 15th of last month, (July, 1842,) when, while the patient was under the effects of a cathartic of gamboge and jalap, a *third* discharge occurred *viâ recti et vaginæ.* The matter now discharged very much resembled that formerly passed in the same manner, but was much more offensive—so much so that I never saw it—and was about the same in quantity. The patient, for four or five days after, complained of a continual "gurgling" sensation in the bowels, and twice threw from the stomach matter resembling, as she said, that just passed from the rectum and vagina. For two consecutive days—and no longer, though the weather was very warm—a healthy perspiration made its appearance—a circumstance before unknown for ten whole years. The patient is now able to move about and attend to her domestic duties—not very arduous, as she has never been pregnant.

She complains of considerable pain over the left ovary, which can be felt in its usual situation. This pain seems materially relieved by the Unguentum Ext. Belladonnæ. The size of the ovary is about that of a common butternut.

Bowels regular; pulse weak, languid, and at times remitting. There is at present also considerable pain down the left limb, attended with much weakness of the part.

Perhaps I might mention that, for a few days before the last disappearance of the tumour the pain from it was considerably increased during and after the passage of urine. Was this occasioned by the withdrawal of the support afforded to the tumour by a distended vesica urinaria?

Such is the history of the case, given as briefly as possible. The question suggests itself, Can any means relieve this patient by preventing the reappearance of this tumour? Upon this point I would invite the opinions of the profession.

*Broome Co., N. Y.*, August, 1842.

